# Comparison of Ultem 9085 Used in Fused Deposition Modelling (FDM) with Polytherimide Blends

**DOI:** 10.3390/ma11020285

**Published:** 2018-02-12

**Authors:** Gianluca Cicala, Giulia Ognibene, Salvatore Portuesi, Ignazio Blanco, Mario Rapisarda, Eugenio Pergolizzi, Giuseppe Recca

**Affiliations:** 1 Dipartimento di Ingegneria Civile e Architettura, Università di Catania, Viale Andrea Doria 6, 95125 Catania, Italy; giuliaognibene@live.com (G.O.); salvo.p90@live.it (S.P.); iblanco@dii.unict.it (I.B.); rapisardamario@yahoo.com (M.R.); euper@hotmail.com (E.P.); 2 UdR-Catania Consorzio National Interuniversity Consortium of Materials Science and Technology (INSTM), Viale Andrea Doria 6, 95125 Catania, Italy; 3 Institute for Polymers, Composites and Biomaterials (ICPB), National Research Council (CNR), Via Paolo Gaifami 17, 95125 Catania, Italy; giuseppe.recca@cnr.it

**Keywords:** thermoplastic blends, additive manufacturing, fused deposition modelling, mechanical properties

## Abstract

Polyetherimide (PEI) blends modified by either polycarbonate (PC) or polyethylene terephthalate glycol-modified (PETG) were prepared. The latter modifier (PETG) was an industrial grade widely used for fused deposition modelling (FDM) printing. PEI blends were compared to Ultem 9085, which is the standard PEI grade for FDM printing in advanced applications. All the blends were thoroughly characterized in terms of their rheological, morphological, thermomechanical and tensile properties. Ultem 9085 showed improved rheology for processing over standard PEI. PEI/PC blends with 10 wt % of modifier developed here closely matched the viscosity behavior of Ultem 9085. On the other hand, the blends with low PC content (i.e., less than 20 wt %) outperformed Ultem 9085 in terms of thermal and tensile properties. When PETG was added, similar tensile properties to Ultem 9085 were found. The immiscibility for PC contents higher than 20 wt % deteriorated the tensile properties, making it less attractive for applications, although melt viscosity decreased further for increasing PC contents.

## 1. Introduction

Additive manufacturing (AM) is gaining increasing importance in industry not only for prototyping, but, in many cases, for the production of functional parts [1]. The global market for AM products and services has grown into a $1.3 billion industry, and it is estimated to grow to over $5 billion by 2020 [1]. Among the different AM techniques, the filament based technology referred as the Fused Deposition Modelling (FDM) is the most widely used and it is also recognized to be the best AM technique for functional parts [2]. To further push the application of FDM for functional applications, the development of engineering polymers for FDM is of paramount importance.

Stratasys (Eden Praire, MN, USA) is the leading company that, founded by Scott Crump, developed the original FDM concepts, and it is now producing the reference industrial machine for FDM under the tradename FORTUS. These machines can operate with acrylonitrile butadiene styrene (ABS), nylon (Ny), polycarbonate (PC), PC-ABS, ASA, polyetherimide (PEI), and polyphenylsulfone (PPSF).

Stratsys offers three choices as high performance filaments: Ultem 1010, Ultem 9085 and Polyphenylsulfone (PPSF). Both the Ultem grades are based on polyetherimide (PEI) and they are certified for use in the automotive, medical and aerospace fields. Ultem 1010 is a pure PEI, while Ultem 9085 is reported to be a mixture of PEI and polycarbonate copolymer blend incorporated for improved flow [3]. 

Zaldivar et al. [3] showed recently that Ultem 9085 printed specimen mechanical properties are significantly affected by build orientation. The strength utilization in terms of FDM/injection molded performance can vary from 85.8% for edge printed specimens to 46.5% for up printed samples. This means that the final properties of the FDM printed part are highly anisotropic. The lower performances, compared to injection molded specimens, were the result of the voids in FDM printed parts between fused filaments. Motaparti et al. [4] recently showed that the strength of a thermoplastic interface within FDM part is directly proportional to the intermolecular diffusion across the interface between the fused filaments. The relevance of bond quality between adjacent filaments depends on printing parameters, but, also, on the melt viscosity of the polymer used for the filaments. However, to the best of our knowledge, this aspect has been properly investigated in the literature by a few studies only from McIlroy et al. [5,6].

The aim of the present paper is twofold: to discuss the rheological, morphological and thermomechanical properties of Ultem 9085; to develop laboratory made PEI/PC and PEI/PETG blends, which can compete with Ultem 9085. In order to achieve the latter objective, the properties of the injection molded blends are reported in comparison to pellets obtained from Ultem 9085. 

## 2. Experimental

### 2.1. Materials and Methods

#### 2.1.1. Materials

An industrial grade polycarbonate (PC), named Xantar, was obtained from Mitsubishi Engineering Plastics, Düsseldorf, Germany. An industrial grade polyethylene terephthalate glycol-modified (PETG) branded as XT was purchased as pellets from Colofabb, Belfeld, the Netherlands. XT is a PETG grade specifically developed to be used as filament for FDM printing. An industrial grade polyetherimide (PEI) was purchased from Luvocomm, Hamburg, Germany. For comparison purposes, the filament Ultem 9085 by Stratasys was tested. All of the materials were used as received. 

#### 2.1.2. Blends Compounding and Specimens Manufacturing

PEI based blends were melt mixed in a batch mixer (Brabender 50 EHT, Brabender & Co., Duisburg, Germany) controlled by a Lab-Station. The torque and the temperature were measured during all the tests. The Brabender Software Winmix was used to record the data. The content of PC in the blends varied from 0 wt % to 40 wt %. The blends with PETG were prepared at two PETG contents only, i.e., 5 wt % and 10 wt %. The laboratory-made blends with PEI and PC were dry blended in the desired quantity, and, then, a total of 45 g of the dry blend were introduced in the preheated chamber of the batch mixer at 300 °C. For PEI/PETG blends, the mixer temperature was lowered to 280 °C. The rotor speed was kept at 50 rpm for all the tests. The samples were extracted after 10 min from the loading in the mixer and left to cool at room temperature.

PEI and PEI/PC injection molded specimens were fabricated using a microinjection molder (Megatech H7/18-1, TecnicaDueBi, Fabriano (AN), Italy) with the settings reported in Table 1. The specimens were allowed to cool down in the mold for 5 min before extraction. Ultem 9085 was pelletized and then processed by injection molding to prepare specimens for comparison testing with PEI based blends. Ultem 9085 injection molded specimens were fabricated using a microinjection molder (Megatech H7/18-1) following the injection settings reported in Table 1. Incremental ratios of PC and PETG were added from 5 to 40 wt % and 5 to 10 wt % for PC and PETG, respectively. The choice to add less than 50 wt % of PC is due to the overall lower thermal performances of PC, which can impair the final thermal properties of the blends. Similarly, a lower amount of PETG was added as this polymer has even lower thermal properties than PC. This point is relevant as PEI based filaments are the preferred choice when high glass transition temperature or high head distortion temperature are requirements.

### 2.2. Characterization

#### 2.2.1. Mechanical Testing

Tensile and flexural specimens were prepared accordingly to ASTM D638 [7] and ASTM D790 [8], respectively. The tensile and flexural properties of the printed materials were investigated testing five samples for each kind of the material under study. Tensile properties of printed specimens were measured by using an Instron 5985 universal testing machine (Instron, Milano, Italy), equipped with a load cell of 10 kN. Specimens were tested at a constant speed of 5 mm/min while compliance correction was used. System control and data analysis were performed using Instron′s Blue Hill v.3.

Differences in mechanical results were statistically analyzed by one-way analysis of variance (ANOVA) using Minitab 17 software. To identify which groups were significantly different from other groups, means comparison was done using the Fisher’s test with a 95% confidence level.

#### 2.2.2. Scanning Electron Microscopy (SEM)

Criofractured surfaces were analyzed with an EVO Scanning Electron Microscope (EVO-SEM, Zeiss, Cambridge, UK). An acid mixture (sulfuric acid/distilled water (3:2)) was used to enhance the phase contrast of the PEI/PC blend. The effect of the etching procedure was to increase the contrast between the thermoplastic phases. The etching treatment was carried out by the immersion of the samples in the acid mixture, followed by a stirring time varying from 5 to 20 min. The samples were always washed with water after etching, and were then sputtered with gold. All the samples were gold sputtered up to a thickness of 20 nm by means of a Emitech K-550 sputter coater (Ashford, Kent, UK). An accelerating voltage of 15 kV was used to collect the micrographs. 

#### 2.2.3. Dynamic Mechanical Analysis (DMA)

The viscoelastic behavior of the solid specimens was investigated using a DMA instrument (TRITEC by Triton Technology, City, United Kingdom) by single cantilever geometry and using samples of size (10 × 5 × 2) mm. The tests were carried out at 1 and 10 Hz with a 2 °C/min heating rate ranging from 25 °C to 250 °C.

#### 2.2.4. Rheological Analysis on Polymer Blends

Rheological analyses were performed with a rotational rheometer ARES by TA Instruments (Milan, Italy) equipped with parallel plates of 25 mm diameter. The polymer samples were charged on the pre-heated lower plate of the rheometer, and the upper plate was then lowered to obtain a gap of 1.2 mm. The melting of sample occurred and the excess of the molten polymer was removed with a clean wood spatula. The frequency sweep test was carried out at 350 °C and the frequency varied from 0.1 to 100 rad/s. The strain was fixed at 5% because some preliminary runs, at various frequencies, showed linear viscoelastic behavior of the blends in these experimental conditions.

## 3. Results and Discussion

### 3.1. Rheological Properties of Ultem 9085 versus PEI Based Blends 

**Figure 1:**
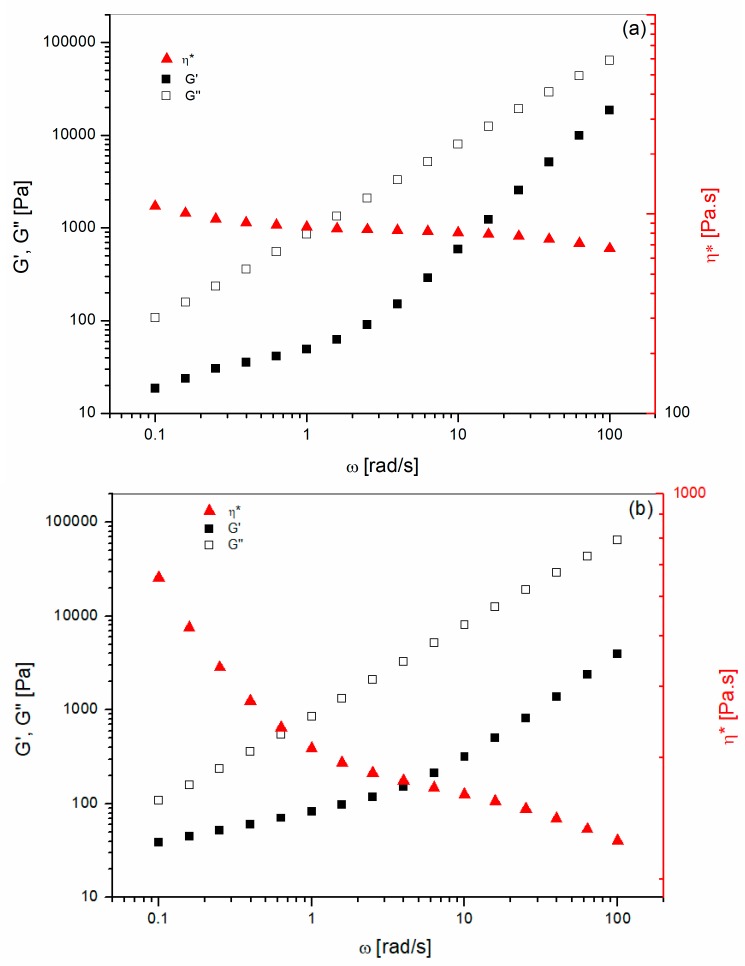
Isothermal rheology at 350 °C for: (**a**) PEI; (**b**) Ultem 9085.

The rheological properties of the pure blend′s components were analyzed first. The linear viscoelastic properties of pure polymers have been investigated using frequency sweep measurements with low amplitude strain (i.e., 5%). The complex viscosity (η*), the storage modulus (G′) and the loss modulus (G′′) as a function of frequency for pure PEI and Ultem 9085 are showed in Figure 1. For both polymers, the complex viscosity decreases with increasing frequencies. However, Ultem 9085 (Figure 1a) presented a lower viscosity compared to unmodified PEI over the entire frequency range. In addition to that, Ultem 9085 displayed a markedly shear thinning behavior compared to the unmodified PEI. The complex viscosity versus frequency of the unmodified PEI used in this study resembled the behavior reported by Nobile et al. [9] for standard Ultem 1000. 

**Figure 2:**
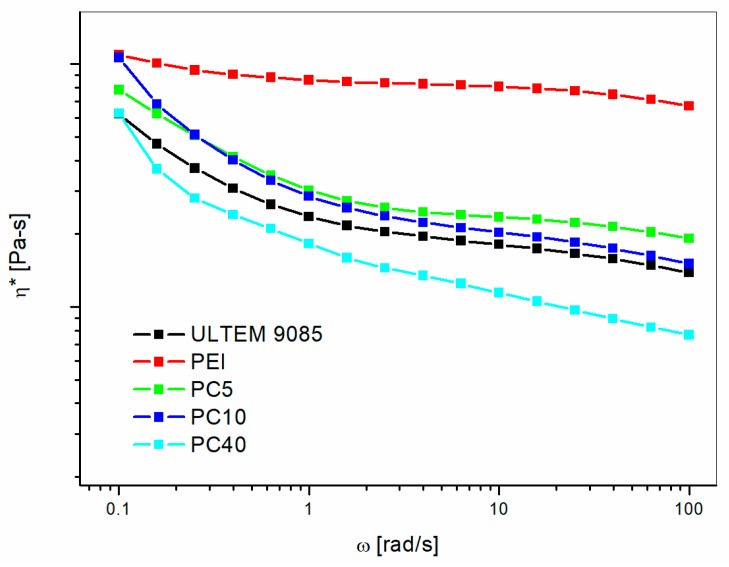
Isothermal rheology at 350 °C for PEI/PC blends with different content of PC.

The complex viscosities for the modified PEI blends with different PC contents are displayed in Figure 2. 

**Figure 3:**
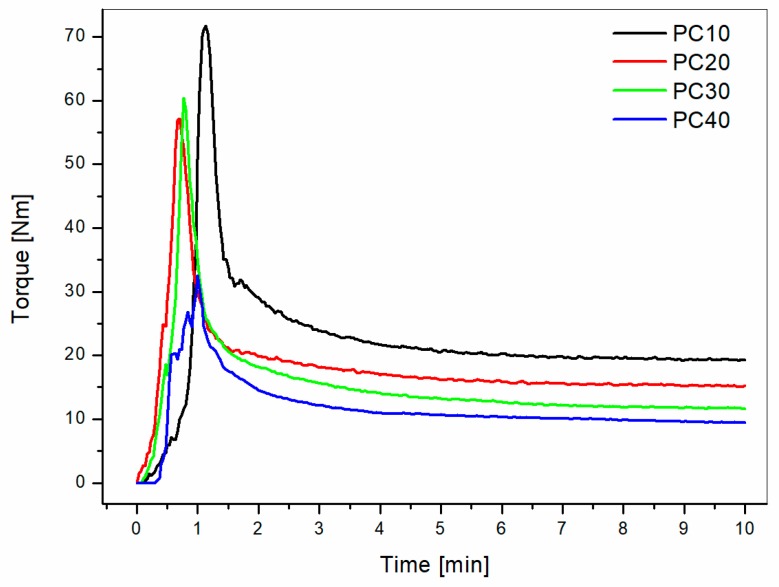
Torque versus time curves for PEI/PC blends mixed at 300 °C for different PC contents.

The graph shows that adding PC or PETG resulted in decreasing viscosities with increasing modifier contents in the high frequencies range. Nobile et al. [9] showed, adding thermotropic liquid crystalline polymer, that the viscosity of PEI is lowered by the inclusion of a low viscosity second phase. Similar results were obtained by Ramiro et al. [10] by measuring the exit melt pressure directly from the extruder for PEI modified by Poly(trimethyleneterephatalate) (PTT). All of these findings support the conclusion that adding PC or PETG leads to improvements in melt processing of PEI based blends. These results were further confirmed by the torque measured during melt blending in the static mixer (Figure 3). 

The clear effect of PETG to lower the processing window of PEI was demonstrated by the preparation of mixed blends at 280 °C in the static mixer, while injection molded samples were manufactured at 320 °C instead of 350 °C that was used for PEI/PC. 

### 3.2. Dynamic Mechanical Analysis of Ultem 9085 versus PEI Based Blends

**Figure 4:**
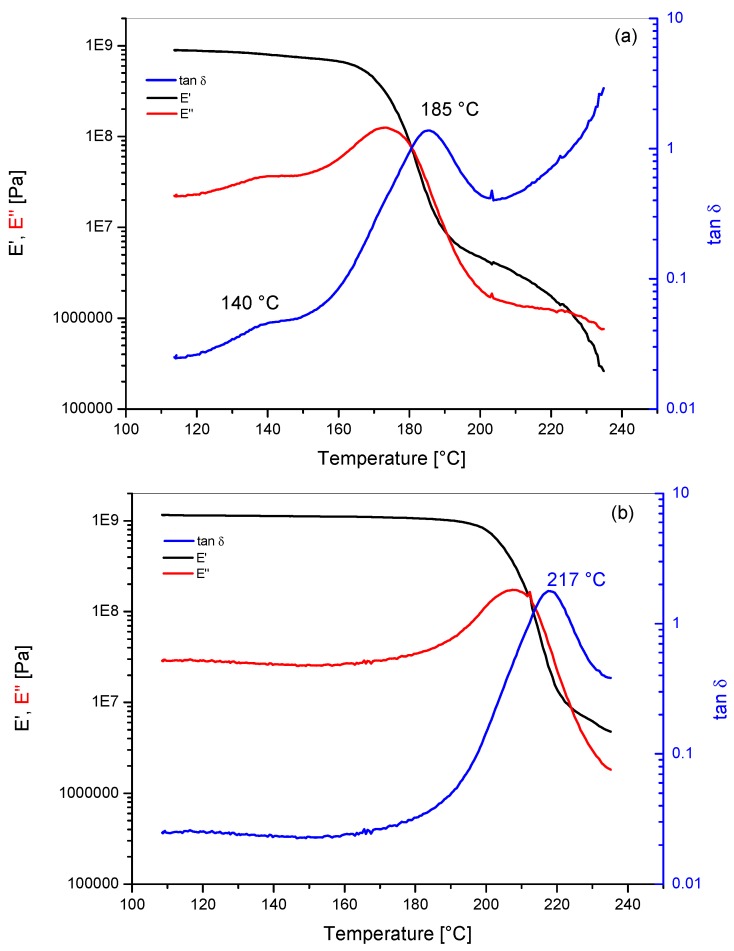
Dynamic mechanical analysis for PEI based systems: (**a**) Ultem 9085 and (**b**) PEI.

Storage modulus (E′), loss modulus (E′′) and loss factor (Tanδ) versus temperature plots for the unmodified PEI and for Ultem 9085 are reported in Figure 4. The unmodified PEI showed a single clear Tanδ peak centered at 217 °C, while, for Ultem 9085, a wide peak at 185 °C and a shoulder at 140 °C were observed. The presence of this shoulder for Ultem 9085 could be the result of the presence of a second phase dispersed in the polymer as stated by Zaldivar et al. [3]. Similar behavior was observed in the loss modulus curves. The unmodified PEI showed the standard behavior reported for PEI industrial grades like Ultem 1000, while similar shifts to lower temperatures for the main Tanδ peak were reported previously for blends PEI mixed with compatibilized second phase [10]. 

**Figure 5:**
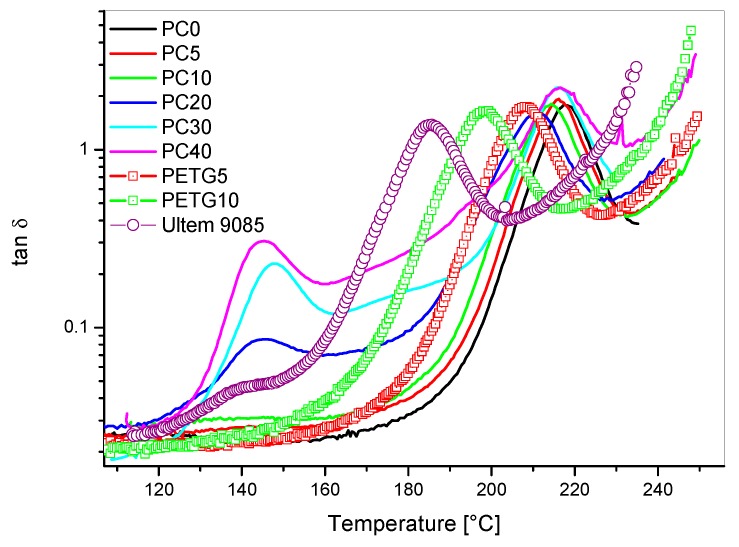
Tan δ versus temperature for PEI/PC and PEI/PETG blends compared to Ultem 9085: effect of PC and PETG content.

**Figure 6:**
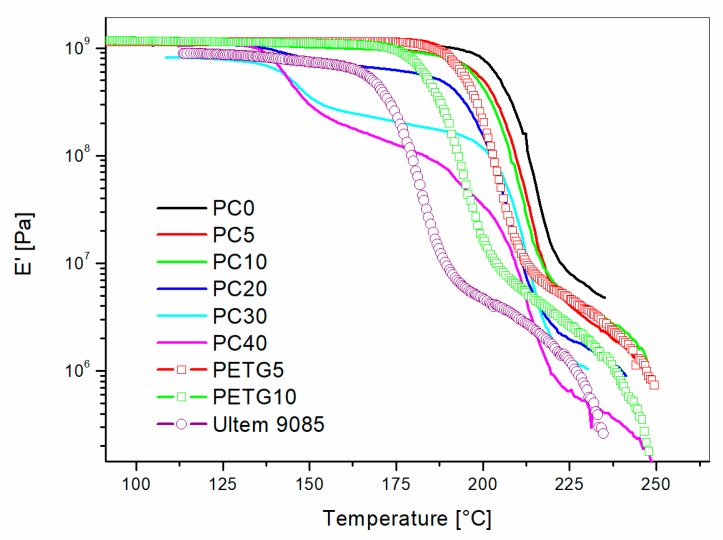
Storage Moudulu (E′) versus temperature for PEI/PC and PEI/PETG blends compared to Ultem 9085: effect of PC and PETG content.

**Figure 7:**
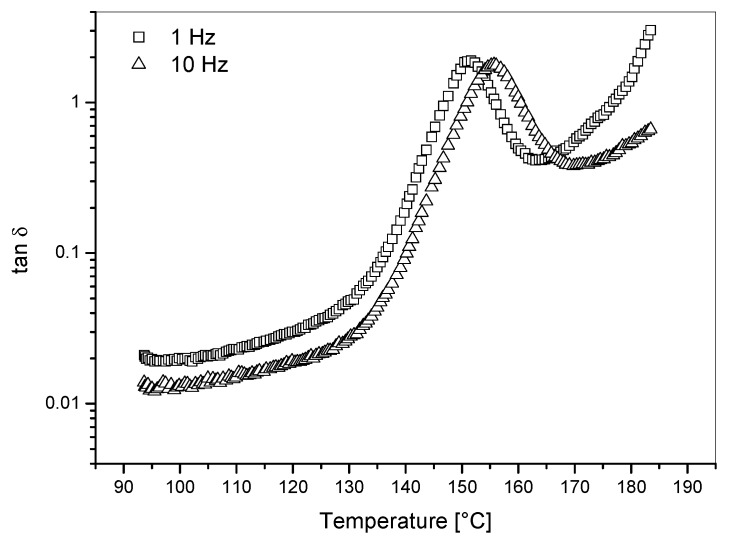
Tan δ versus temperature for PC used as modifier in PEI/PC blends tested at two different frequencies (i.e., 1 Hz and 10 Hz).

The effects on Tanδ and storage modulus (E′) of blending PC or PETG with PEI are showed in Figure 5 and Figure 6, respectively. The Tanδ curves showed two distinct peaks for PEI/PC blends with PC content higher than 10 wt %: one main peak centered between 210 °C and 217 °C; a smaller Tanδ peak centered between 143 °C and 148 °C. Pure polycarbonate (PC) showed one single Tanδ peak at 150 °C in correspondence to its glass transition temperature (Figure 7). The blends modified with PETG showed one relaxation peak only but for decreasing temperatures with increasing PETG content.

**Figure 8:**
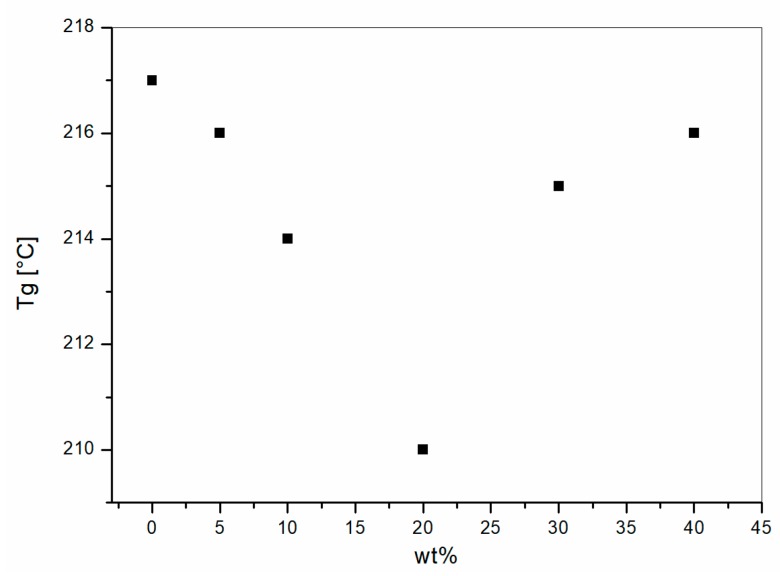
Glass Transition Temperature (Tg) versus PC content for the PEI rich phase (data extrapolated from peak analysis of Figure 6).

The presence of two Tanδ peaks in the blends with PC content higher than 10 wt % was the result of the immiscibility between PEI and PC. The shift of Tg’s for PEI-rich phases in the PEI/PC blends is reported as a function of PC weight content in Figure 8. 

The glass transition trend for different PC content, which were observed previously [11,12], can be explained by the presence of residual PC dispersed in the PEI-rich phase at high PEI compositions. However, the shift measured are smaller than those observed for PEI/PETG. In addition to that, PEI/PETG blends showed one single Tanδ peak for both the PETG concentrations analyzed, thus leading to the conclusion that PETG had an enhanced miscibility with PEI compared to PC. Several authors demonstrated that PEI is miscible with PET and PBT polymers [12,13]; therefore, PETG could be a miscible polymer with PEI.

**Figure 9:**
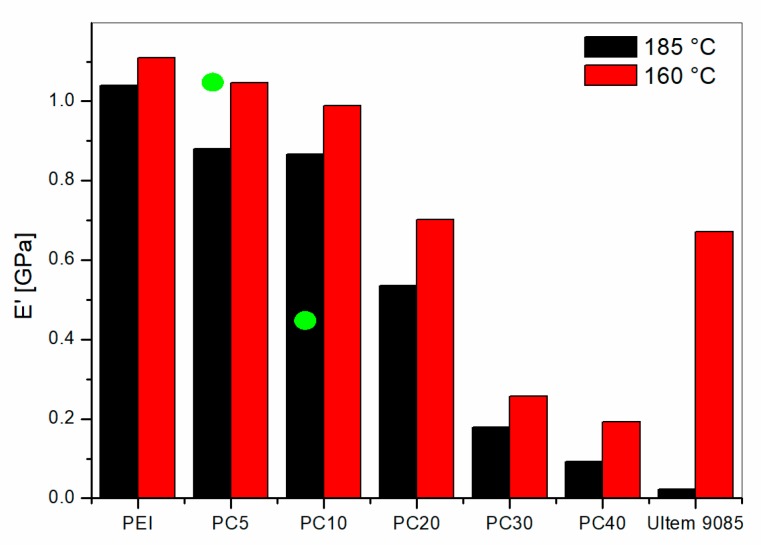
Storage Modulus values calculated at 165 °C and 185 °C from graph of E′ versus temperature. Green circles represent the value for PEI/PETG blends.

The comparison of the storage modulus (E′′) versus temperature for all the PEI/PC blends showed clear modulus drops for temperatures lower than 175 °C for the blends with PC content higher than 20 wt %. The blends with 5 wt % and 10 wt % of PC showed modulus drops only for temperatures higher than 190 °C. The E′ values measured at 160 °C and 185°C are reported in Figure 9 for all the PEI/PC blends and for the Ultem 9085. 

PEI/PC blends displayed higher storage modulus than for Ultem 9085 at 185 °C. However, for the measurements at 160 °C, the blends with PC contents up to 10 wt % showed similar or higher storage modulus compared to Ultem 9085. These systems, which showed one Tanδ peak only, were characterized by a phase separation that did not allow for the formation of PC-rich phases, greatly impacting the storage modulus of the blends. The PEI/PC with enhanced lower Tanδ peaks showed higher modulus drops at 160 °C, which lead to a smaller modulus compared to Ultem 9085. The blend PC20 showed a transitional behavior with modulus at 160 °C similar to Ultem 9085. Those results were further supported by the microscopic investigation of the fractured surface reported below.

PEI/PETG blends showed a single modulus drop. The storage modulus values at 185 °C for PETG5 and PETG10 were 1.03 and 0.46 GPa, respectively. These values are displayed as green circles in Figure 9 for comparison with PEI/PC blends. The graph shows that, for low PETG content (i.e., 5 wt %), the modulus of the blend is higher compared to the PC5, but, as the PETG content is increased up to 10 wt %, the modulus was halved compared to that of PC10. This difference was due to the increased content of PETG dispersed homogenously in the PEI matrix for PETG10 blend. However, if the comparison is made between PEI/PETG and Ultem 9085, the storage modulus at 185 °C is always higher for the PETG modified blends. 

### 3.3. Morphological Analysis of Ultem 9085 versus PEI/PC Blends

**Figure 10:**
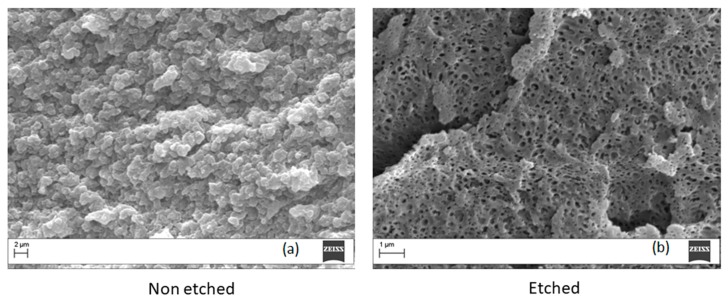
Micrographs of crio-fractured Ultem 9085 samples before (**a**) and after (**b**) chemical etching (magnification 20,000×).

The morphology of PEI/PC blends was studied on criofractured surfaces using a scanning electron miscroscopy (SEM). The micrographs of Ultem 9085 are shown in Figure 10 for both etched and non-etched specimens. No peculiar morphology is displayed on the non-etched surface (Figure 10a). For this reason, we performed an acid etching on the surface of the sample and the result is shown in Figure 10b. The analysis on the etched surface revealed a particulate morphology with a well dispersed phase separation of small (about 250 nm) particles. This result is coherent with the observed Tanδ curve, which showed a main peak and a shoulder at low temperature. 

**Figure 11:**
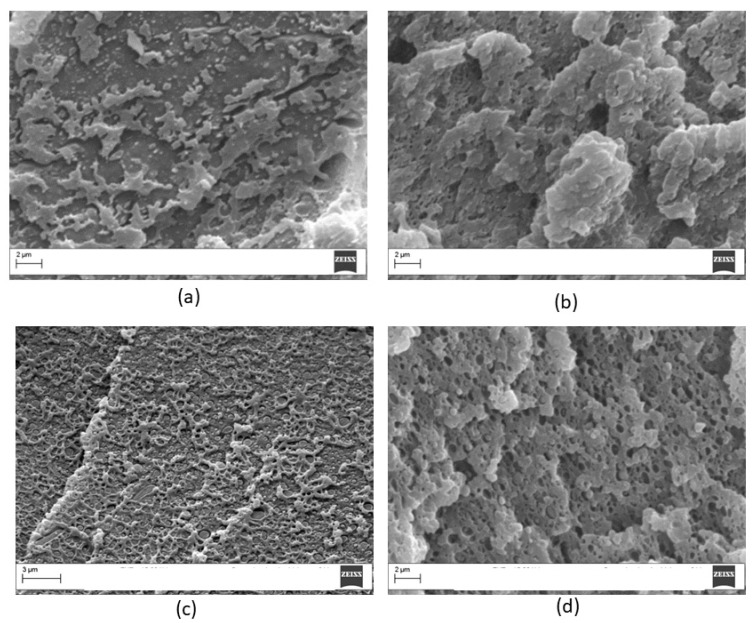
Micrographs of crio-fractured PEI/PC blends containing 20 wt % of PC for different etching time (magnification 10,000×): (**a**) 5 min; (**b**) 10 min; (**c**) 15 min; (**d**) 20 min.

Similar analysis was carried out on the PEI/PC blend with 20 wt % of PC and the results, for different etching times, are shown in Figure 11. The blend showed clear phase separation for all of the etched surfaces. However, after 20 min of etching, the presence of particulate was clearer. In order to obtain enhanced phase contrast, all of the PEI modified blends were analyzed applying the etching procedure. 

**Figure 12:**
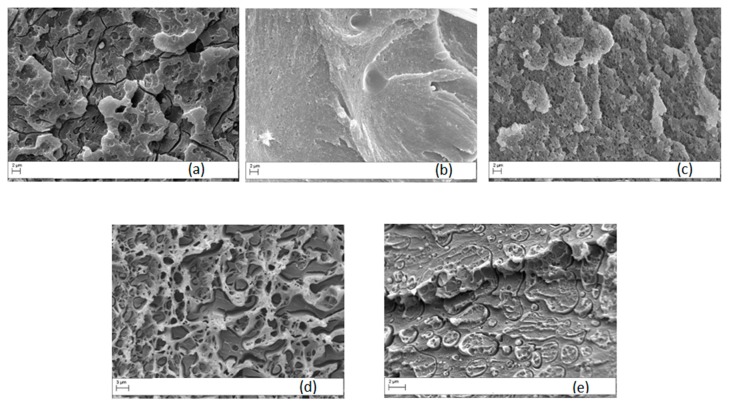
Micrographs of crio-fractured PEI/PC blends for different PC content (magnification 10,000×): (**a**) 5 wt %; (**b**) 10 wt % ; (**c**) 20 wt % ; (**d**) 30 wt % ; (**e**) 40 wt %.

**Figure 13:**
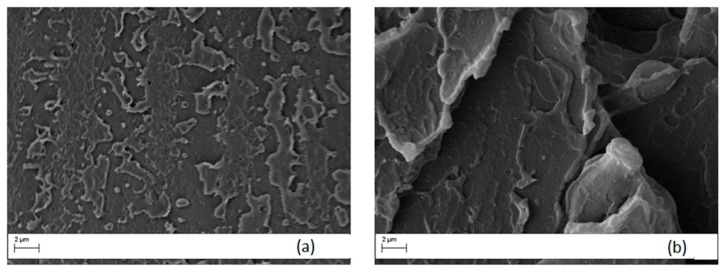
Micrographs of crio-fractured PEI/PETG blends for different PETG content (magnification 10,000×): (**a**) 5 wt %; (**b**) 10 wt %.

Figure 12 reports the morphology of the blends with different contents of PC while Figure 13 shows the SEM analysis on PETG modified blends. 

The results of the SEM analysis confirmed the findings of DMA described above because, for PC content below 20 wt %, no clear phase separation was observed at the resolution analyzed. When the PC content increased up to 20 wt %, particulate morphology with PC-rich particles of about 0.3 µm appeared. The blend with 30 wt % of PC showed a co-continuous morphology, while phase inversion was observed when PC content was 40 wt %. In contrast to this behavior, when PETG was used as a modifier, morphologies similar to Ultem 9085 were observed as the result of the improved miscibility of PETG and PEI (Figure 13). 

### 3.4. Mechanical Properties of Ultem 9085 versus PEI/PC Blends 

**Figure 14:**
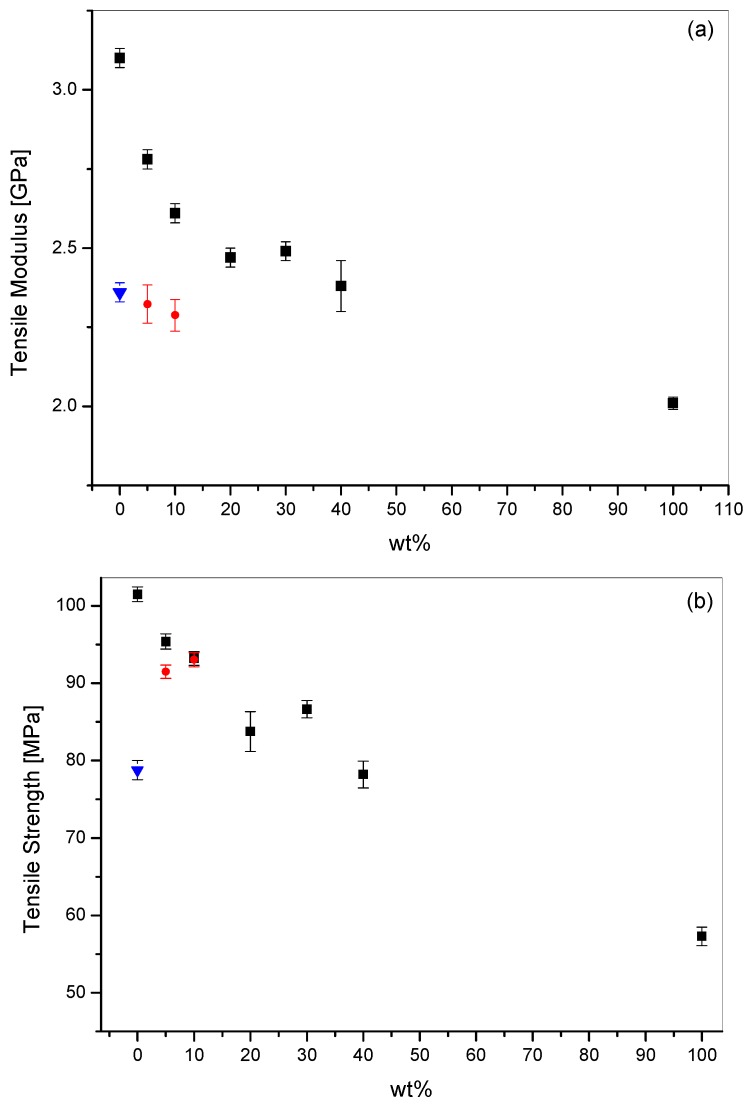
Effect of modifier content for PEI/PC (black) and PEI/PETG (red) blends compared to Ultem 9085 (blue) on tensile modulus (**a**) and tensile strength (**b**).

The tensile properties for all the PEI blends and for Ultem 9085 were determined at room temperature. The results are summarized in Figure 14 for the tensile modulus and tensile yield strength. Ultem 9085 displayed a tensile modulus and a yield strength of 2.36 ± 0.03 GPa and 78.77 ± 0.94 MPa, respectively. These values were higher than those reported in the technical literature for Ultem 9085 by Stratasys, but this is in accordance to what presented previously when comparing injection molded specimens to FDM samples [14]. The unmodified PEI used here showed tensile modulus and yield strength of 3.10 ± 0.03 GPa and 101.49 ± 0.94 MPa, respectively. The values found are higher for unmodified PEI because it is a pure PEI rather than a blend as for Ultem 9085. 

The PEI/PC blends showed a decreasing trend for both the modulus and strength with increasing values of PC content. However, for PC content up to 40 wt %, the PEI/PC blends showed equal or higher tensile properties compared to Ultem 9085 samples. The experimental data for both tensile modulus and strength showed negative deviations from the rule of mixture. Ramiro et al. [15] showed similar behavior for PEI/PC blends, which were interpreted as the result of the slightly different orientation level of the components in the blends and in the pure state. Similar behavior is observed in the blends prepared here, which showed analogous morphologies to those reported by Ramiro et al. [15].

ANOVA analysis helped to shed more light on the influence of PC content. The tensile modulus means were all significantly different (*p* < 0.05) with the exception of the pairwise 20–30 (*p* = 0.333). The yield strength means seemed, accordingly to Fisher, not significantly different for the pairwise 0–5, 5–10 and 20–40. However, if the measured values were directly compared, only the 20–40 showed *p*-value higher than 0.05 (0.728), thus confirming that the means are not statically different. The ANOVA analysis on the PEI/PETG blends revealed that the tensile modulus and yield strength of the systems with 5 wt % and 10 wt % were not statistically different with *p*-value of 0.307 and 0.809, respectively.

## 4. Conclusions

PEI based blends were prepared by melt mixing adding two different modifiers: PC and PETG. The PEI blends properties were measured and compared to Ultem 9085, which is a qualified PEI grade used by the leading company Stratasys for FDM. 

Ultem 9085 showed a different viscosity behavior when compared to standard unmodified PEI. The viscosity for Ultem 9085 was lower thus with improved melt processing. Adding PC and PETG had similar effects on the viscosity leading to decreasing viscosity for increasing modifier contents. PC, in particular, when added at 10 wt % showed a complex behavior similar to Ultem 9085. Therefore, the findings presented here demonstrated that PEI based blends with processing properties comparable to Ultem 9085 can be easily obtained by mixing standard PEI with a selected amount of PC. 

DMA analysis showed a distinct different behavior for PEI modified by PC and PETG. The latter reduced the blend′s glass transition temperature of 10 °C and 20 °C when 5 wt % and 10 wt % of modifier were added, respectively. SEM highlighted a homogenous morphology, which lead to the conclusion that miscible blends were formed upon mixing. Ultem 9085 showed high Tg reduction for the main phase, but, in this case, a small shoulder appeared at 140 °C and small particles were observed by SEM, which leads to the conclusion that this system is a compatibilized blend. On the contrary, PEI/PC blends showed glass transition temperature reductions of maximum 10 °C for the blend with 20 wt % of PC. In addition to that, two single peaks were clearly showed for the blends with PC content higher than 10 wt % and SEM analysis confirmed the presence of biphasic morphologies for these blends. The phase separation was not clearly observed for PEI/PC blends with 5 wt % and 10 wt % of PC. 

The tensile properties and the storage modulus at 160 °C and 185 °C provided evidence that PEI blends with PC content lower than 20 wt % of PC behaved better than Ultem 9085 for all of the parameters considered. In terms of tensile yield strength, PEI/PC and PEI/PETG behaved similarly, but PEI/PC outperformed PETG based blends in terms of the tensile modulus. For PC content higher than 20 wt %, the PEI/PC lost its advantages over Ultem 9085 showing higher storage modulus at 185 °C only. 

Ultem 9085 filament is sold by Stratasys with 1.5 kg cartridge costing about 750 €. From other filament producers [16] Ultem 9085 is sold at about 200 €/kg. However, unmodified ultem, such as the grade used in this paper, can be purchased in pellets at 50 €/kg only, while PC is purchased at 20 €/kg. The data reported in this paper provided evidence that standard PEI can be modified with 10 wt % of PC to obtain a blend with promising properties to compete with Ultem 9085 in terms of similar melt processing properties but overall better performances. In addition to that, adding PC to PEI can further reduce the cost per kilogram, and, in turn, reduce by at least half the final price of the filaments.

## Figures and Tables

**Table 1:** Polymer blend ratios prepared by injection molding alongside with the parameters for processing.

**CODE**	**PEI**	**PC**	**T1**	**T2**	**T3**	**T4**	**Pressure**
	**(wt%)**	**(wt%)**	**(°C)**	**(°C)**	**(°C)**	**(°C)**	**(bar)**
PC100	0	100	220	280	300	40	30
PC0	100	0	320	350	350	60	90
PC5	95	5	320	350	350	60	90
PC10	90	10	320	350	350	60	90
PC20	80	20	320	350	350	60	90
PC30	70	30	320	350	350	60	90
PC40	60	40	320	350	350	60	90
	PEI Stratasys		300	330	330	60	90
	**PEI**	**PETG**	**T1**	**T2**	**T3**	**T4**	**Pressure**
	**(wt%)**	**(wt%)**	**(°C)**	**(°C)**	**(°C)**	**(°C)**	**(bar)**
PETG5	95	5	320	330	330	60	90
PETG10	90	10	320	330	330	60	90
